# Supplementary material for: ECMPride: prediction of human extracellular matrix proteins based on the ideal dataset using hybrid features with domain evidence
Source: PeerJ. 2020 Apr 29;8:e9066. doi: 10.7717/peerj.9066 (PMC7195829; doi:10.7717/peerj.9066)
Supplement: Table S5 — When the prediction model is built without the under-sampling ensemble method, the model gains a high specificity of 0.9995, but the sensitivity is very low, and also present a poor balanced accuracy of 0.7579. When the under-sampling ensemble method is used, the model achieves a balance between the specificity (0.9360) and sensitivity (0.8925) and gains a high balanced accuracy of 0.9142 as well. Therefore, the under-sampling ensemble method can solve the problem of imbalance of the dataset well and makes full use of the sample information at the same time. [file peerj-08-9066-s005.docx]

**Table S5:**

**Performance of the prediction model with or without the under-sampling ensemble method.**

When the prediction model is built without the under-sampling ensemble method, the model gains a high specificity of 0.9995, but the sensitivity is very low, and also present a poor balanced accuracy of 0.7579. When the under-sampling ensemble method is used, the model achieves a balance between the specificity (0.9360) and sensitivity (0.8925) and gains a high balanced accuracy of 0.9142 as well. Therefore, the under-sampling ensemble method can solve the problem of imbalance of the dataset well and makes full use of the sample information at the same time.

| Method | Sensitivity | Specificity | Accuracy | Balanced accuracy |
| --- | --- | --- | --- | --- |
| With ensemble | 0.8925 | 0.9360 | 0.9340 | 0.9142 |
| Without ensemble | 0.5163 | 0.9995 | 0.9782 | 0.7579 |
