# Supplementary figures and images for: ECMPride: prediction of human extracellular matrix proteins based on the ideal dataset using hybrid features with domain evidence
Source: PeerJ. 2020 Apr 29;8:e9066. doi: 10.7717/peerj.9066 (PMC7195829; doi:10.7717/peerj.9066)

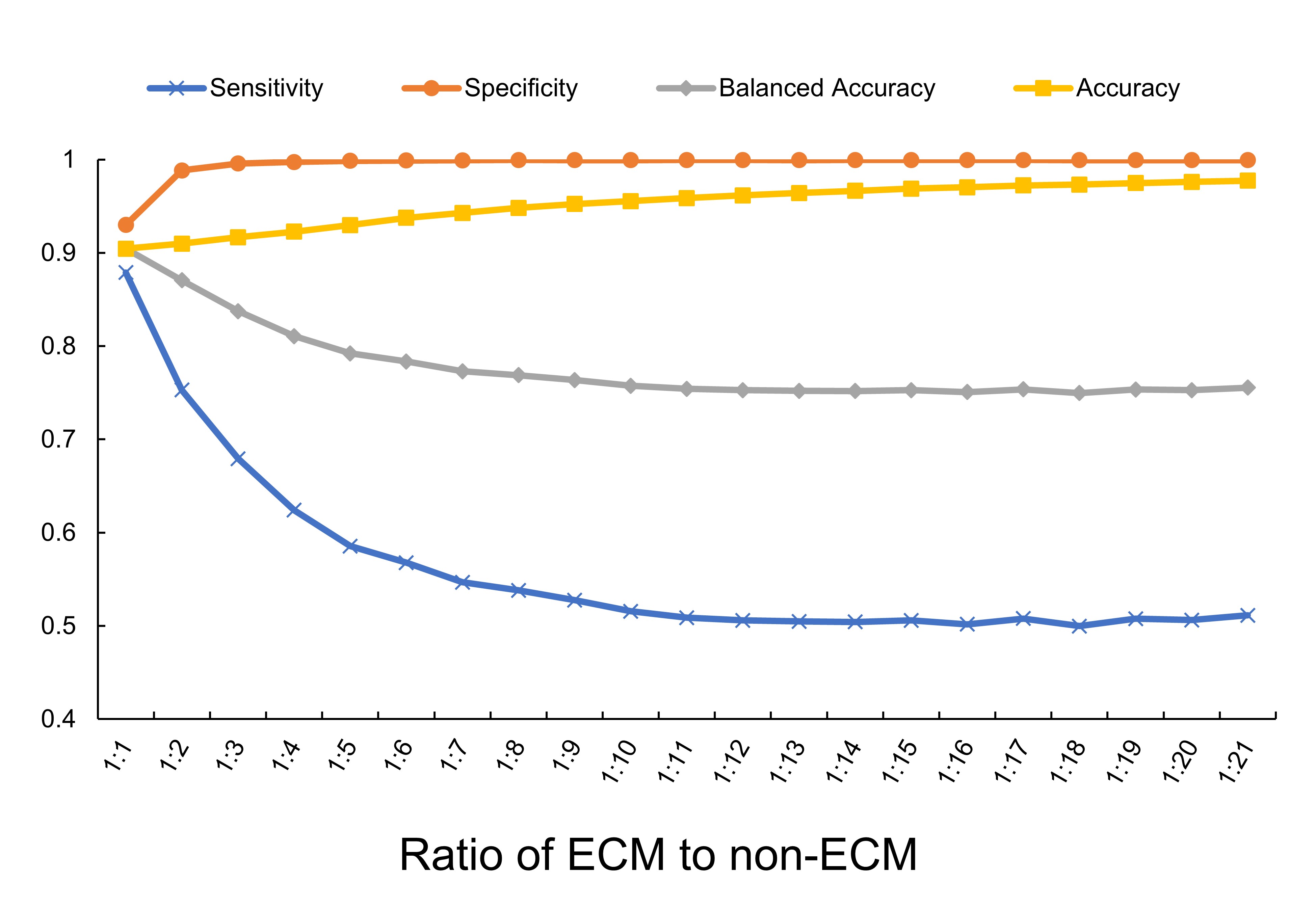

Supplement: Figure S1 — As the ratio of ECM to non-ECM samples in the training dataset is approximately 1:21, we decide to construct 21 training datasets, with the ratio of ECM cases to non-ECM cases from 1:1 to 1:21. In each dataset, ECM cases are the entire ECM dataset and non-ECM cases are randomly selected from the non-ECM dataset. Then, the prediction model is implemented on these 21 different imbalance datasets separately using 10-fold cross-validation, and four performance assessment parameters are calculated, including Sensitivity, Specificity, Accuracy and balanced Accuracy. The above process is repeated 10 times, and the average performance result of 10 times was taken as the final result. As the ratio of non-ECMs to ECMs increases, the specificity increases gradually, while the sensitivity decreases significantly, which indicates a declining trend of model performance. At the same time, the Balanced Accuracy decreases along with the increasing of imbalance ratio, indicates this parameter can better represent the model performance than Accuracy. [file peerj-08-9066-s009.jpg]

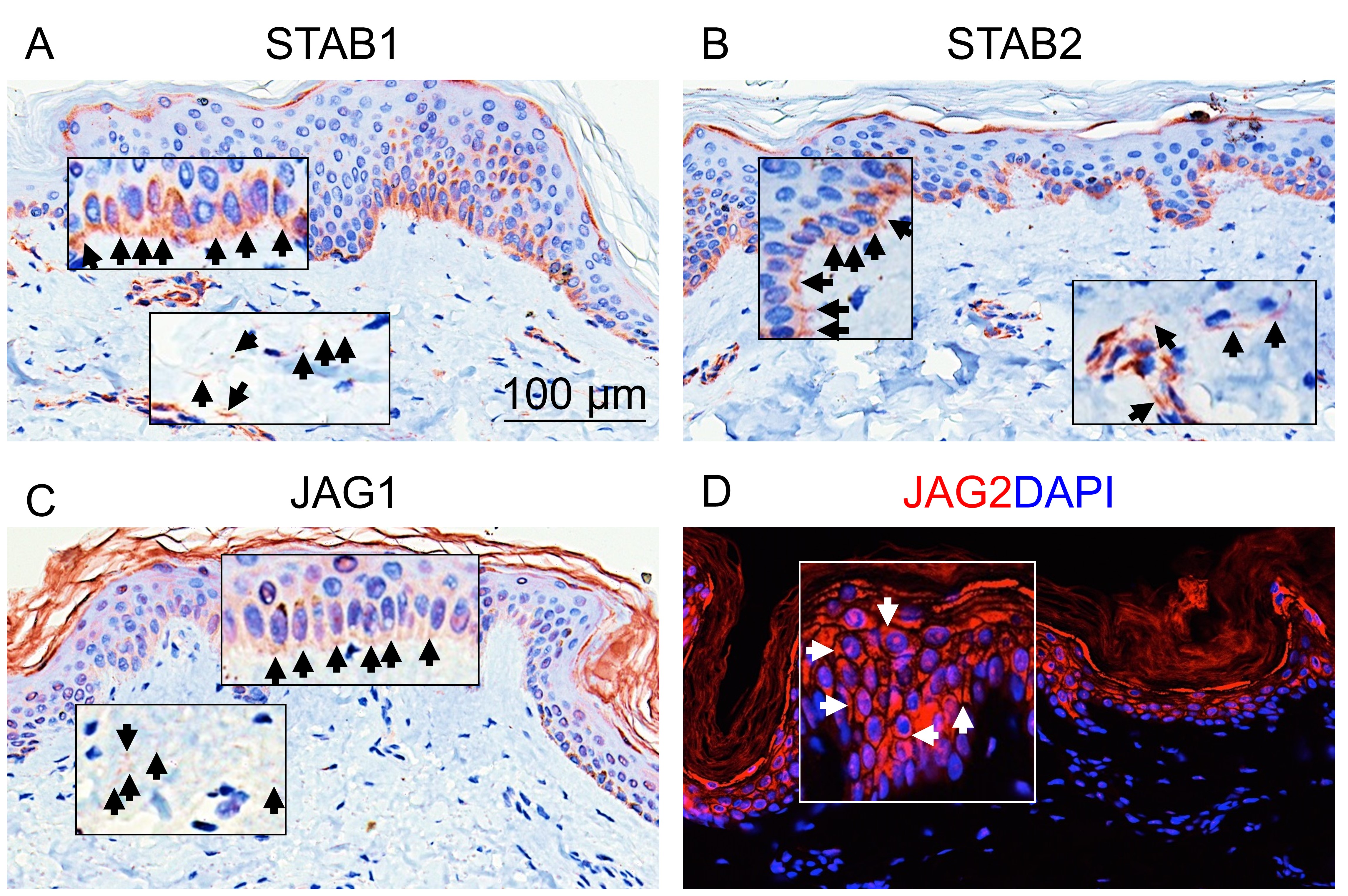

Supplement: Figure S2 — Immunohistochemistry and immunofluorescence analysis of (A) STAB1, (B) STAB2, (C) JAG1 and (D) JAG2 on normal human skin tissues (scale bar: 100 μm). Black and white arrowheads pointed to the expressed locations of the new ECM proteins. [file peerj-08-9066-s010.jpg]

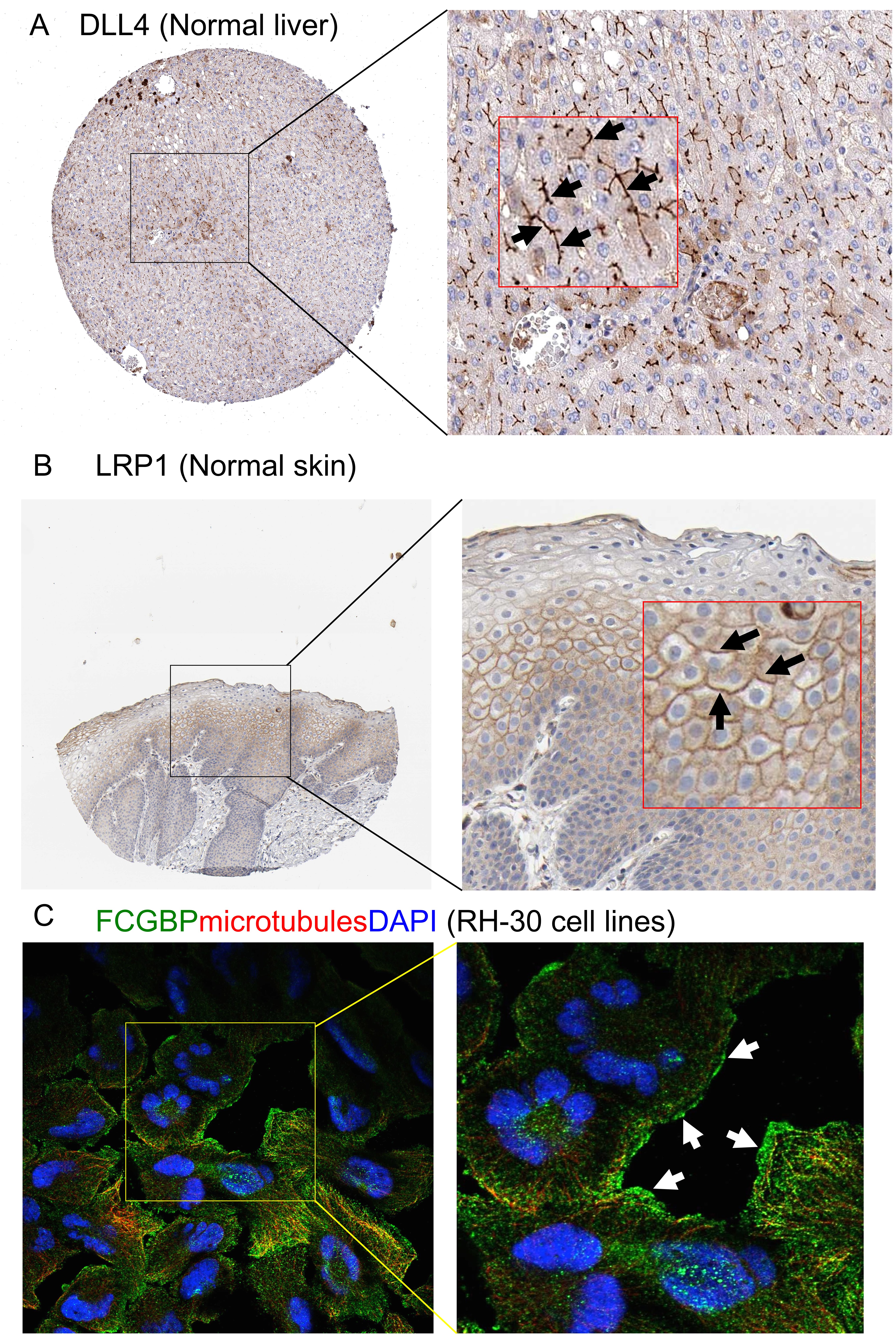

Supplement: Figure S3 — Immunohistochemistry analysis of (A) DLL4 and (B) LRP1 on human normal liver and skin tissues. (C) Immunofluorescence analysis of FCGBP on RH-30 cell lines. Black and white arrowheads pointed to the expressed locations of the new ECM proteins. The data comes from https://www.proteinatlas.org database website. [file peerj-08-9066-s011.jpg]
