# Supplementary material for: ECMPride: prediction of human extracellular matrix proteins based on the ideal dataset using hybrid features with domain evidence
Source: PeerJ. 2020 Apr 29;8:e9066. doi: 10.7717/peerj.9066 (PMC7195829; doi:10.7717/peerj.9066)
Supplement: Article S1 [file peerj-08-9066-s012.docx]

**Introduction of existing ECM prediction tools**

ECMPP is a long-established tool for predicting ECM proteins through machine learning modeling; when introduced, it was groundbreaking in the field of ECM protein prediction (Jung et al. 2010). In the construction of standard datasets, although researchers subsequently extensively used the standard dataset developed by EcmPred (Kandaswamy et al. 2013), the construction of EcmPred’s dataset is identical to that of ECMPP: they both select metazoan secreted protein sequences from SwissProt as the initial dataset and then select those proteins with an ECM annotation as ECM components and proteins without ECM annotation as non-ECM components; the final standard dataset is formed after the elimination of redundancy. Therefore, we believe that ECMPP is also a pioneering approach that provides a strategy for constructing the standard dataset of the ECM protein prediction tool.

The most significant contribution of EcmPred is the construction of a standard dataset widely used by researchers on subsequent ECM protein prediction tools (Kandaswamy et al. 2013). However, there is a problem of imbalance in the dataset: the number of samples in the positive dataset (445) is much smaller than that in the negative dataset (4,187). If an unbalanced dataset is used as the training set, the predictive accuracy of the small sample dataset (the positive dataset) will deteriorate. In response to this problem, Kandaswamy *et al.* proposed a solution to randomly select the same number of ECM samples (300) and non-ECM samples (300) from the original dataset into the training set, thus making the sizes of the negative dataset and positive dataset in the training set the same. This method avoids the problem of poor predictive accuracy of the model in the positive dataset, but prevents full use of the sample information of the original dataset, and can only be used as a preliminary solution.

PECM used the PSSM feature for the first time and adopted the SVM classification algorithm (Zhang et al. 2014). Compared to the general characteristics, the batch extraction of PSSM requires the use of specific tools (such as PSI-BLAST) and appropriate background libraries (such as SwissProt) on the one hand (Consortium 2017), and it also takes more time (The length of time is proportional to the size of the background library). However, in practice, it has been proven that these costs are worthwhile because PSSM is, indeed, an essential feature for predicting ECM proteins and was widely used in the development of subsequent ECM protein prediction tools. One of the characteristics of SVM is that the two parameters C and γ have a great influence on the model, so parameter adjustment can be applied to significantly improve the performance of the model. Researchers generally use the grid search strategy to adjust parameters (Kabir et al. 2018; Zhang et al. 2014).

IECMP introduces balanced accuracy (BAcc) to measure the performance of predictive models while using integrated classifiers to deal with the problem of imbalance in the dataset (Yang et al. 2015). In the balanced dataset, accuracy can better reflect the overall performance of the model. However, since the number of non-ECM proteins in the standard dataset far exceeds that of ECM proteins, the model will be more inclined to judge a sample as not being a component of the ECM, resulting in a model with high accuracy and low sensitivity, so that accuracy can’t reflect the overall performance of the model well. Therefore, in an unbalanced dataset, BAcc better reflects the overall performance of the model than accuracy.

IECMP also proposed the idea of the under-sampling ensemble method, which solves the problem of imbalance of the dataset well and makes full use of the sample information at the same time. First, the negative dataset in the training set is randomly divided into 11 negative subsets, so that the number of each negative subset is close to the positive dataset in the training set. Then, 11 ECM protein prediction models are constructed using these 11 negative subsets and the positive dataset as training sets. Finally, the 11 prediction models are used to predict and vote on the samples in the testing dataset, and the majority of each testing sample is used as the final prediction result of the testing sample.

ECMP-HybKNN builds an effective predictive tool with easy-to-extract features (Ali & Hayat 2016). It chooses dipeptide composition (DPC) and pseudo-amino acid composition (PseAAC) as the extraction features. The extraction of these two features is relatively simple, and the computational efficiency is also improved accordingly.

As the first of its kind, TargetECMP builds an ECM protein prediction tool using only one classification feature and achieves better prediction results (Kabir et al. 2018). In the past, ECM protein prediction tools often integrated multiple features to reflect more differentiated information. TargetECMP only used evolutionary information extracted from the grey system model (GreyPSSM) to obtain significantly good performance of the model. This good performance may occur for the following reasons: (I) GreyPSSM is, indeed, a superior ECM protein prediction feature, and (II) TargetECMP improves model performance by adjusting the parameters for the SVM classifier.

Starting with EcmPred, these ECM prediction tools all use the same dataset, so parameters such as sensitivity and specificity of the model can be directly compared. As time goes on, the sensitivity and specificity of tools tend to be more balanced, and the balanced accuracy of tools gradually improves. The performance comparison of existing ECM prediction tools is shown in Table S1-1, including the major performance evaluation features sensitivity, specificity, balanced accuracy, as well as some features about the tools.

Table S1-1. The comparison of existing ECM prediction tools

| **Tool name** | ECMPP | EcmPred | PECM | IECMP | ECMP-HybKNN | BAMORF | TargetECMP |
| --- | --- | --- | --- | --- | --- | --- | --- |
| **Release date** | 2010 | 2013 | 2014 | 2015 | 2016 | 2017 | 2018 |
| **Dataset** | Jung | Kandaswamy | Kandaswamy | Kandaswamy | Kandaswamy | Kandaswamy | Kandaswamy |
| **Extracted features** | SI/EI | SI/PP | EI/SI/PP | SI/PP/EI/SI | SI | SI/PP | EI |
| **Feature selection method** | Mean Decrease Accuracy | Maximum Relevance Minimum Redundancy | Fisher–Markov selector | Information Gain Ratio | Maximum Relevance Minimum Redundancy | Binary animal migration | -- |
| **Machine learning method** | Random Forest | Random Forest | Support vector machine | Random Forest | K nearest neighbor | Random Forest | Support vector machine |
| **Cross-validation method** | 5-fold cross validation | Independent dataset test | Independent dataset test | 10-fold cross validation | 10-fold cross validation | Independent dataset test | Jackknife test |
| **Sensitivity (%)** | 56.3 | 65.0 | 75.9 | 87.8 | 84.2 | 85.0 | 93.1 |
| **Specificity (%)** | 99.2 | 77.0 | 86.9 | 84.9 | 97.8 | 86.5 | 94.2 |
| **Balanced accuracy (%)** | 77.8 | 71.0 | 81.4 | 86.4 | 91.0 | 85.7 | 93.7 |
| **Accuracy (%)** | 95.6 | 77.0 | 86.5 | 85.1 | 96.8 | 86.5 | 94.1 |
| **Whether to provide the tool** | Yes | Yes | Yes | Yes | No | No | No |
| **Availability of the tool** | No | No | No | No | No | No | No |
| **Whether the tool can be reproduced** | No | Yes | No | No | No | No | No |

Jung: The standard dataset used by Jung et al. to build ECMPP in 2010

Kandaswamy: The standard dataset used by Kandaswamy et al. to build EcmPred in 2013

SI: Extracted features based on the sequence information

EI: Extracted features based on the evolutionary information

SI: Extracted features based on the structural information

PP: Extracted features based on physicochemical properties

Among all prediction tools introduced above, four of them (ECMPP, EcmPred, PECM and IECMP) were released with web-based applications when the papers were published. Unfortunately, none of these tools are currently available. We tried to rebuild these models according to the literatures, only EcmPred can be constructed probably.

Also, only EcmPred was applied to predict all human entries and got 2201 putative ECM proteins (Kandaswamy et al. 2013). We converted these protein IDs into gene symbols and compared them with Matrisome. In all 2,201 putative ECMs predicted by ECMPred, only 70 of them were included in Matrisome (core matrisome: 22, matrisome-associated: 48), while the overlap between the putative ECMs predicted by ECMPride and Matrisome is 715 (Figure 3, core matrisome: 253, matrisome-associated: 462). These results suggest that ECMPride could predict more reliable ECM components compared with EcmPred.

**Construction of the standard dataset**

**The positive dataset:** Genes in Matrisome with further credible evidence were selected to form the positive dataset. The credible evidence is mainly that these genes are detected in healthy tissues of people. To this end, we firstly selected genes in ECMatlas (Naba et al. 2016) which derived from healthy tissues and added them to the positive dataset. Afterwards, we collected more ECM proteomic studies in healthy tissues (especially tissues not included in ECMatlas, such as bone and teeth) and obtained the corresponding ECM lists (Information of collected studies are listed in Table S2), genes in these lists that coincide with Matrisome were also added to the positive dataset.

For Matrisome genes not detected in healthy tissues, we believe those that are detected in disease tissues or in core Matrisome also very likely to be true ECM components. Therefore, we firstly collected these genes (the method collecting Matrisome genes detected in disease tissues is the same as above), and then mined the GO annotations of them (Consortium 2016). Genes containing annotation of “extracellular matrix” were considered to be true ECM and were eventually added to the positive dataset. Finally, we converted ECM genes to SwissProt IDs via UniProt and obtained 521 ECM proteins to form the positive dataset. Also, the composition figure of this positive dataset is shown in Figure S1-1, showing the number of genes comprising each Matrisome category.


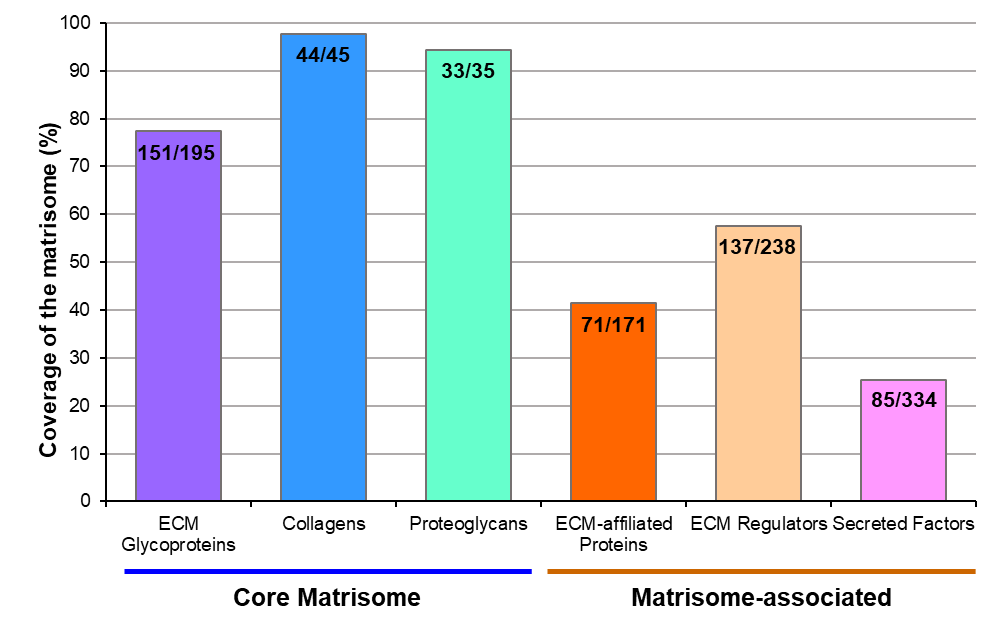


**Figure S1-1. Positive dataset coverage of the Matrisome.** Bar chart represents, for each Matrisome category, the percentage representation and number of ECM genes included in the positive dataset.

**The negative dataset:** Thul *et al.* divided human protein-coding genes into three types: intracellular genes, membrane-spanning genes, and secreted genes, in the Human Protein Atlas (Thul et al. 2017). On the basis of the above classification, the intracellular genes proposed by Thul *et al.* were first collected as non-ECM candidate genes. Second, we screened genes that overlap with Matrisome in non-ECM candidate genes and removed them from the list of non-ECM candidate genes to form the list of non-ECM genes. Finally, we converted non-ECM genes to SwissProt IDs via UniProt and obtained 11,336 non-ECM proteins to form the negative dataset.

**Conversion process of PSSM**

The initial PSSM is shown in formula (1)

$$\begin{aligned} P_{PSSM}=\left[ \begin{matrix} \begin{matrix} E_{1, 1} & E_{1, 2} \\ E_{2, 1} & E_{2, 2} \end{matrix} & \begin{matrix} \cdots& E_{1, j} \\ \cdots& E_{2, j} \end{matrix} & \begin{matrix} \cdots& E_{1, 20} \\ \cdots& E_{2, 20} \end{matrix} \\ \begin{matrix} \vdots& \vdots\\ E_{i, 1} & E_{i, 2} \end{matrix} & \begin{matrix} \cdots& \vdots\\ \cdots& E_{i, j} \end{matrix} & \begin{matrix} \cdots& \vdots\\ \cdots& E_{i, 20} \end{matrix} \\ \begin{matrix} \vdots& \vdots\\ E_{L, 1} & E_{L, 2} \end{matrix} & \begin{matrix} \cdots& \vdots\\ \cdots& E_{L, j} \end{matrix} & \begin{matrix} \cdots& \vdots\\ \cdots& E_{L, 20} \end{matrix} \end{matrix} \right]\#\left( 1 \right) \end{aligned}$$

Where $E_{i, j}$ represents the score of the amino acid mutation in the *i*-th position of the sequence to form the amino acid type *j* during evolution. First, we standardize every element in the original matrix as follows:

$$\begin{aligned} E_{i, j}^{1}=\frac{E_{i, j} - \overline{E_{i}}}{SD\left( \overline{E_{i}} \right)}\#\left( 2 \right) \end{aligned}$$

Here, $E_{i, j}$ represents the original values of $P_{PSSM}$, and $\overline{E_{i}}$ and $SD(\overline{E_{i}})$ represent the average and the standard deviation of the values in the *i*-th row of the matrix $P_{PSSM}$, respectively. Therefore, the matrix $P_{PSSM}$ is converted into $P_{PSSM}^{1}$:

$$\begin{aligned} P_{PSSM}^{1}=\left[ \begin{matrix} \begin{matrix} E_{1, 1}^{1} & E_{1, 2}^{1} \\ E_{2, 1}^{1} & E_{2, 2}^{1} \end{matrix} & \begin{matrix} \cdots& E_{1, j}^{1} \\ \cdots& E_{2, j}^{1} \end{matrix} & \begin{matrix} \cdots& E_{1, 20}^{1} \\ \cdots& E_{2, 20}^{1} \end{matrix} \\ \begin{matrix} \vdots& \vdots\\ E_{i, 1}^{1} & E_{i, 2}^{1} \end{matrix} & \begin{matrix} \cdots& \vdots\\ \cdots& E_{i, j}^{1} \end{matrix} & \begin{matrix} \cdots& \vdots\\ \cdots& E_{i, 20}^{1} \end{matrix} \\ \begin{matrix} \vdots& \vdots\\ E_{L, 1}^{1} & E_{L, 2}^{1} \end{matrix} & \begin{matrix} \cdots& \vdots\\ \cdots& E_{L, j}^{1} \end{matrix} & \begin{matrix} \cdots& \vdots\\ \cdots& E_{L, 20}^{1} \end{matrix} \end{matrix} \right]\#\left( 3 \right) \end{aligned}$$

Then, the matrix $P_{PSSM}^{1}$ is compressed into a vector $V_{20}$ of length 20:

$$\begin{aligned} V_{20}=\left[ \begin{matrix} \overline{E_{1}} & \overline{E_{2}} \end{matrix}, \cdots,\begin{matrix} \overline{E_{19}} & \overline{E_{20}} \end{matrix} \right]\#\left( 4 \right) \end{aligned}$$

where

$$\begin{aligned} \overline{E_{j}}=\frac{1}{L}\sum_{i=1}^{L} E_{i, j}^{1} \left( j=1, 2, \ldots, 20 \right)\#\left( 5 \right) \end{aligned}$$

In addition to the vector $V_{20}$, we also extract a vector $V_{60}$ of length 60 via a grey system approach. According to grey system theory (Chou 2001; Matsuda et al. 2005), there is a “white system” whose information is fully known, along with a “black system” whose information is completely unknown and a “grey system” whose information is partially known. When faced with problems of insufficient or uncertain information, the model based on the grey system theory is particularly useful (Lin et al. 2013).

The vector obtained by the grey system can be expressed as

$$\begin{aligned} V_{60}=\left[ \begin{matrix} \lambda_{1} & \lambda_{2} \end{matrix}, \cdots,\begin{matrix} \lambda_{3j-1} & \lambda_{3j} \end{matrix} \right] \left( j=1, 2,\ldots, 19, 20 \right)\#\left( 6 \right) \end{aligned}$$

where

$$\begin{aligned} \left[ \begin{matrix} \lambda_{3j-2} \\ \lambda_{3j-1} \\ \lambda_{3j} \end{matrix} \right]=\left( B_{j}^{T}B_{j} \right)^{-1}B_{j}^{T}U_{j} \left( j=1, 2, \ldots, 19, 20 \right)\#\left( 7 \right) \end{aligned}$$

where

$$\begin{aligned} B_{j}=\left[ \begin{matrix} {-E}_{2, j}^{1} & \left( -E_{1, j}^{1}-0.5E_{2, j}^{1} \right) & 1 \\ {-E}_{3, j}^{1} & \left( -\sum_{i=1}^{2} E_{i, j}^{1}-0.5E_{3, j}^{1} \right) & 1 \\ \begin{matrix} \vdots\\ {-E}_{L, j}^{1} \end{matrix} & \begin{matrix} \vdots\\ \left( -\sum_{i=1}^{L-1} E_{i, j}^{1}-0.5E_{L, j}^{1} \right) \end{matrix} & \begin{matrix} \vdots\\ 1 \end{matrix} \end{matrix} \right]\#\left( 8 \right) \end{aligned}$$

and

$$\begin{aligned} U_{j}=\left[ \begin{matrix} E_{2, j}^{1}-E_{1, j}^{1} \\ E_{3, j}^{1}-E_{2, j}^{1} \\ \begin{matrix} \vdots\\ E_{L, j}^{1}-E_{L-1, j}^{1} \end{matrix} \end{matrix} \right]\#\left( 9 \right) \end{aligned}$$

Finally, an 80-D feature vector of PSSM is obtained, which combines $V_{20}$ and $V_{60}$ for every protein sequence.

**Maximum Relevance Minimum Redundancy (mRMR)**

mRMR is largely based on the terms of mutual information (MI), which is one of the methods widely used to define the relevance of variables (Peng et al. 2005). The MI of variables x and y is calculated as follows:

$$\begin{aligned} I\left( x,y \right)=\int\int p(x,y)log\frac{p\left( x,y \right)}{p\left( x \right)p\left( y \right)}dxdy\#\left( 1 \right) \end{aligned}$$

where$p\left( x \right)$,$p(y)$, and $p(x,y)$ are their probabilistic density functions.

In practice, we can use the incremental search methods of mRMR to find the near-optimal features while scoring the importance of different features. Supposing that there is a feature set *F*, we already select a subset $F_{s}$ containing m features, and there remains a subset $F_{r}$ containing n features (${F=F}_{s} + F_{r}$). For a feature $f_{i}$ in $F_{r}$, its relevance with class c can be calculated as follows:

$$\begin{aligned} D_{i}=I\left( f_{i},c \right)\#\left( 2 \right) \end{aligned}$$

The redundancy of the feature $f_{i}$ in $F_{r}$ with all features in $F_{s}$ can be calculated as follows:

$$\begin{aligned} R_{i}=\frac{1}{m}\sum_{f_{j}\in F_{s}} I\left( f_{i},f_{j} \right)\#\left( 3 \right) \end{aligned}$$

The score of the feature $f_{i}$ with relevance minus redundancy is

$$\begin{aligned} {DR}_{i}&=D_{i}-R_{i} \\ &=I\left( f_{i},c \right)-\frac{1}{m}\sum_{f_{j}\in F_{s}} I\left( f_{i},f_{j} \right)\#\left( 4 \right) \end{aligned}$$

Among all features in $F_{r}$, the feature $f_{i}$ with maximum relevance minimum redundancy can be found using the following formula:

$$\begin{aligned} max\left[ {DR}_{i} \right]=\begin{matrix} max \\ f_{i}\in F_{r} \end{matrix}\left[ I\left( f_{i},c \right)-\frac{1}{m}\sum_{f_{j}\in F_{s}} I\left( f_{i},f_{j} \right) \right]\#\left( 5 \right)\# \end{aligned}$$

For a feature set containing M features, we can obtain a ranked feature set by executing the above process for M rounds.

**Cross-validation and under-sampling ensemble method**

Ten-fold cross-validation is used to train the model, and the dataset is first randomly divided into 10 subsets on average. Next, we take turns to choose one subset to become a testing dataset, and the others are used as a training dataset. Finally, 10 combinations of training dataset and testing dataset are obtained. In each run, we use the training dataset to train the model and the testing dataset to test the model’s performance; we take the average result of 10 runs as the final result of the model.

The under-sampling ensemble method is performed in the following two steps:

**Step 1.** In each run of the 10-fold cross-validation, non-ECM proteins are much more than ECM proteins in the training dataset. In order to balance the number of ECM proteins and non-ECM proteins, we randomly select 99 groups of non-ECM proteins in the training dataset to form 99 non-ECM subsets, the number of non-ECM proteins in each of which is equal to the number of ECM proteins in the training dataset. Then we combine each subset of non-ECM proteins with ECM proteins to become a training subset. Finally, we obtain 99 training subsets in which the numbers of ECM and non-ECM proteins are the same.

**Step 2.** On the basis of the 99 training subsets obtained above, we train 99 models with Random Forest. Then, the testing dataset of each run is predicted by each of the 99 models, and the final predicted result is determined by the majority votes among the 99 models.

**The calculation of performance evaluation parameters**

Sensitivity refers to the proportion of samples in the positive dataset correctly predicted as being components of the ECM:

$$\begin{aligned} Sn=\frac{TP}{TP + FN} \end{aligned}$$

Specificity refers to the proportion of samples in the negative dataset that are correctly predicted as not being components of the ECM:

$$\begin{aligned} Sp=\frac{TN}{TN + FP} \end{aligned}$$

Accuracy refers to the proportion of correct predictions of the components and non-components of the ECM in the dataset:

$$\begin{aligned} Acc=\frac{TP + TN}{TP + FN + TN + FP} \end{aligned}$$

Balanced accuracy refers to the mean of sensitivity and specificity:

$$\begin{aligned} BAcc=\frac{Sn + Sp}{2} \end{aligned}$$

**Histology and immunofluorescence experiment validation**

Human foreskin tissues from patients undergoing circumcision were obtained from the Pathology biosample bank in Chinese PLA General Hospital; all samples were stored at −80°C prior to analysis. And the study was approved by the local ethics committee. Samples were fixed in 4 % formaldehyde overnight at 4 °C and then processed for gradient dehydration. After tissues embedded in paraffin, the sections were cut at 4 μm thickness for immunohistochemistry and immunofluorescence analysis. Briefly, sections were blocked with Avidin/Biotin Blocking Kit, stained with anti-STAB1 (YN2255, ImmunoWay), STAB2 (YN2164, ImmunoWay), JAG1 (70109, Cell Signaling Tech), JAG2 (ab109627, Abcam) antibodies overnight at 4 °C. After washing in PBS, sections were incubated for 1 hour at room temperature with secondary antibodies. DAPI was stained for 12 minutes. At last, sections were sealed with Fluoro-Gel for Photographic. Each negative control samples were incubated with secondary antibody alone. The pictures were taken at 20× /40× magnification, and analyzed by Volocity Demo (× 64).

As shown in Fig S2, STAB1, STAB2, JAG1 and JAG2 are all expressed in the extracellular space of epidermis and dermis. Interestingly, STAB1, STAB2 and JAG1 specifically expressed in the basement membrane (BM) of skin tissue, which is composed of ECM components and plays an important role in the polarity and differentiation of basal stem cells as an epidermal stem cell niches (Jones et al. 2007). The presentation of experimental interactions with known ECM proteins could be supportive evidence for the new putative ECMs. The protein-protein interactions of the 779 putative ECMs uniquely predicted by ECMPride with ECMs in Matrisome are retrieved both from MatrixDB (Clerc et al. 2018) and STRING (Szklarczyk et al. 2018) database. As shown in Table S7, a total of ten known ECMs were found interacted with STAB1 or STAB2. Interestingly, SPARC is recognized as an important proteoglycan expressed on BM, which affect cell function and ECM remodeling through the release of soluble factors by MMPs enzyme (Sage et al. 2003). Previous studies have proved that SPARC and STAB1 have direct interaction and STAB1 can act as the receptor of SPARC (Kzhyshkowska et al. 2006; Workman & Sage 2011). Besides, STAB1 mediated SPARC uptake and endosomal trafficking to regulate the concentration of extracellular SPARC (Kzhyshkowska et al. 2006). JAG1 and JAG2 also have interactions with several known ECMs, most of which are important cytokines (also ECM secreted factors), including EGF, TGFB1, FGFs, BMPs, interleukins, WNTs, etc. (Table S7).

**References**

**Ali F, and Hayat M. 2016.** Machine learning approaches for discrimination of Extracellular Matrix proteins using hybrid feature space. *Journal of Theoretical Biology* **403**:30-37.

**Chou KC. 2001.** Prediction of protein cellular attributes using pseudo‐amino acid composition. *Proteins: Structure, Function, and Bioinformatics* **43**:246-255.

**Clerc O, Deniaud M, Vallet SD, Naba A, Rivet A, Perez S, Thierry-Mieg N, and Ricard-Blum S. 2018.** MatrixDB: integration of new data with a focus on glycosaminoglycan interactions. *Nucleic Acids Research* **47**:D376-D381.

**Consortium GO. 2016.** Expansion of the Gene Ontology knowledgebase and resources. *Nucleic Acids Research* **45**:D331-D338.

**Consortium U. 2017.** UniProt: the universal protein knowledgebase. Nucleic Acids Research **45**:D158-D169.

**Jones PH, Simons BD, and Watt FM. 2007.** Sic transit gloria: farewell to the epidermal transit amplifying cell? *Cell Stem Cell* **1**:371-381.

**Jung J, Ryu T, Hwang Y, Lee E, and Lee D. 2010.** Prediction of extracellular matrix proteins based on distinctive sequence and domain characteristics. *Journal of Computational Biology* **17**:97-105.

**Kabir M, Ahmad S, Iqbal M, Swati ZNK, Liu Z, and Yu D-J. 2018.** Improving prediction of extracellular matrix proteins using evolutionary information via a grey system model and asymmetric under-sampling technique. *Chemometrics and Intelligent Laboratory Systems* **174**:22-32.

**Kandaswamy KK, Pugalenthi G, Kalies K-U, Hartmann E, and Martinetz T. 2013.** EcmPred: Prediction of extracellular matrix proteins based on random forest with maximum relevance minimum redundancy feature selection. *Journal of Theoretical Biology* **317**:377-383.

**Kzhyshkowska J, Workman G, Cardó-Vila M, Arap W, Pasqualini R, Gratchev A, Krusell L, Goerdt S, and Sage EH. 2006.** Novel function of alternatively activated macrophages: stabilin-1-mediated clearance of SPARC. *The Journal of Immunology* **176**:5825-5832.

**Lin W-Z, Fang J-A, Xiao X, and Chou K-C. 2013.** iLoc-Animal: a multi-label learning classifier for predicting subcellular localization of animal proteins. *Molecular BioSystems* **9**:634-644.

**Matsuda S, Vert JP, Saigo H, Ueda N, Toh H, and Akutsu T. 2005.** A novel representation of protein sequences for prediction of subcellular location using support vector machines. *Protein Science* **14**:2804-2813.

**Naba A, Clauser KR, Ding H, Whittaker CA, Carr SA, and Hynes RO. 2016.** The extracellular matrix: Tools and insights for the “omics” era. *Matrix Biology* **49**:10-24.

**Peng H, Long F, and Ding C. 2005.** Feature selection based on mutual information: criteria of max-dependency, max-relevance, and min-redundancy. *IEEE Transactions on Pattern Analysis & Machine Intelligence*:1226-1238.

**Sage EH, Reed M, Funk SE, Truong T, Steadele M, Puolakkainen P, Maurice DH, and Bassuk JA. 2003.** Cleavage of the matricellular protein SPARC by matrix metalloproteinase 3 produces polypeptides that influence angiogenesis. *Journal of Biological Chemistry* **278**:37849-37857.

**Szklarczyk D, Gable AL, Lyon D, Junge A, Wyder S, Huerta-Cepas J, Simonovic M, Doncheva NT, Morris JH, and Bork P. 2018.** STRING v11: protein–protein association networks with increased coverage, supporting functional discovery in genome-wide experimental datasets. *Nucleic Acids Research* **47**:D607-D613.

**Thul PJ, Åkesson L, Wiking M, Mahdessian D, Geladaki A, Blal HA, Alm T, Asplund A, Björk L, and Breckels LM. 2017.** A subcellular map of the human proteome. *Science* **356**:eaal3321.

**Workman G, and Sage EH. 2011.** Identification of a sequence in the matricellular protein SPARC that interacts with the scavenger receptor stabilin‐1. *Journal of Cellular Biochemistry* **112**:1003-1008.

**Yang R, Zhang C, Gao R, and Zhang L. 2015.** An ensemble method with hybrid features to identify extracellular matrix proteins. *PloS One* **10**:e0117804.

**Zhang J, Sun P, Zhao X, and Ma Z. 2014.** PECM: Prediction of extracellular matrix proteins using the concept of Chou’s pseudo amino acid composition. *Journal of Theoretical Biology* **363**:412-418.
